# A novel approach to fake news classification using LSTM-based deep learning models

**DOI:** 10.3389/fdata.2023.1320800

**Published:** 2024-01-08

**Authors:** Halyna Padalko, Vasyl Chomko, Dmytro Chumachenko

**Affiliations:** ^1^Mathematical Modelling and Artificial Intelligence Department, National Aerospace University “Kharkiv Aviation Institute”, Kharkiv, Ukraine; ^2^Ubiquitous Health Technology Lab, University of Waterloo, Waterloo, ON, Canada; ^3^Global Governance Department, Balsillie School of International Affairs, Waterloo, ON, Canada; ^4^System Design Engineering Department, University of Waterloo, Waterloo, ON, Canada

**Keywords:** misinformation, disinformation, fake news, deep learning, LSTM, BiLSTM, attention-based BiLSTM

## Abstract

The rapid dissemination of information has been accompanied by the proliferation of fake news, posing significant challenges in discerning authentic news from fabricated narratives. This study addresses the urgent need for effective fake news detection mechanisms. The spread of fake news on digital platforms has necessitated the development of sophisticated tools for accurate detection and classification. Deep learning models, particularly Bi-LSTM and attention-based Bi-LSTM architectures, have shown promise in tackling this issue. This research utilized Bi-LSTM and attention-based Bi-LSTM models, integrating an attention mechanism to assess the significance of different parts of the input data. The models were trained on an 80% subset of the data and tested on the remaining 20%, employing comprehensive evaluation metrics including Recall, Precision, F1-Score, Accuracy, and Loss. Comparative analysis with existing models revealed the superior efficacy of the proposed architectures. The attention-based Bi-LSTM model demonstrated remarkable proficiency, outperforming other models in terms of accuracy (97.66%) and other key metrics. The study highlighted the potential of integrating advanced deep learning techniques in fake news detection. The proposed models set new standards in the field, offering effective tools for combating misinformation. Limitations such as data dependency, potential for overfitting, and language and context specificity were acknowledged. The research underscores the importance of leveraging cutting-edge deep learning methodologies, particularly attention mechanisms, in fake news identification. The innovative models presented pave the way for more robust solutions to counter misinformation, thereby preserving the veracity of digital information. Future research should focus on enhancing data diversity, model efficiency, and applicability across various languages and contexts.

## 1 Introduction

In the digital age, misinformation has become a pervasive and insidious problem that affects various aspects of society, from politics to public health (Adams et al., 2023). Misinformation refers to any false, inaccurate, or misleading information, regardless of the intention behind its dissemination (Ecker et al., 2022). The rapid advancement of technology and the ubiquity of social media platforms have facilitated the spread of misinformation at an unprecedented rate, making it difficult for individuals to discern fact from fiction (Muhammed and Mathew, 2022). This has led to many negative consequences, including the erosion of trust in institutions (Nahum et al., 2021), the polarization of society (Gupta et al., 2023), and the hindrance of adequate response to crises such as the COVID-19 pandemic (Agley and Xiao, 2021).

A significant subset of misinformation is “fake news,” which refers to false or misleading information presented as news (Zakharchenko et al., 2021). Fake news is often created to deceive, manipulate, or incite and is usually disseminated through online platforms, where it can quickly go viral (van der Linden et al., 2020). The proliferation of fake news has profound implications for democracy and governance, as it can influence public opinion, undermine trust in the media, and exacerbate social divisions (Tenove, 2020). Moreover, fake news can have real-world consequences, such as inciting violence (Hinz et al., 2023) or affecting election outcomes (Mutahi and Kimari, 2020).

The problem of fake news is particularly acute in the context of modern conflicts, such as the Russian war against Ukraine (Pierri et al., 2023). In such situations, both sides often engage in information competition, using strategic communication to shape narratives. Very often bad actors as Russia use fake news and disinformation to manipulate public perception and gain strategic advantage (Bulanova, 2023). This can lead to a distorted understanding of the conflict, hinder diplomatic efforts, and exacerbate tensions. Furthermore, spreading fake news in conflict zones can have dire humanitarian consequences, as it can incite violence, cause panic, and impede aid delivery (Maschmeyer et al., 2023).

Given the grave implications of fake news, there is a pressing need for adequate classification. Automated classification of fake news involves using machine learning algorithms to analyze the content of news articles and determine their veracity (Dasari et al., 2022). This is a challenging task, as fake news is often designed to be convincing and may contain elements of truth. However, natural language processing and machine learning advances have made it possible to develop sophisticated models to classify fake news accurately (Hirlekar and Kumar, 2020). Such models can be integrated into online platforms to flag or filter out fake news in real-time, limiting its spread and mitigating its impact (Zhang et al., 2023).

Deep learning, a subset of machine learning, has shown remarkable effectiveness in detecting fake news (Hu et al., 2022). Deep learning models, particularly neural networks, are capable of processing large amounts of data, extracting intricate patterns, and capturing the nuances of language, which are essential for accurately classifying fake news. These models can analyze the textual content of news articles and other features such as the source, headline, and metadata (Sastrawan et al., 2021). Moreover, deep learning models can be trained to recognize the subtle cues and patterns of fake news, such as sensationalism, bias, and inconsistency (Premanand et al., 2021). As a result, deep learning models have achieved high accuracy in fake news detection, outperforming traditional machine learning models and contributing significantly to the ongoing efforts to combat misinformation.

The aim of the paper is to develop the deep learning model for fake news classification.

To achieve this goal, the following tasks were formulated:

1. To analyze models and methods for fake news classification.
2. To develop the deep learning model for fake news classification based on bidirectional LSTM architecture.
3. To extend the bidirectional LSTM model by incorporating an attention mechanism.
4. To evaluate the models' performance and classification results.

The promising contribution of this paper is significant and multifaceted. It begins with a comprehensive analysis of existing models and methods in fake news classification, providing a solid foundation for developing innovative models. Based on this analysis, the paper introduces two novel deep learning models, one based on bidirectional LSTM architecture and another on attention-based bidirectional LSTM architecture. These models are meticulously designed to capture the complexities and nuances of language characteristic of fake news, thereby enhancing the accuracy and efficiency of fake news classification. A rigorous evaluation of the models' performance and a careful assessment of the classification results provide valuable insights into the effectiveness of the proposed models. Overall, this paper substantially contributes to the ongoing efforts to combat fake news by introducing innovative deep learning models and thoroughly evaluating their performance.

The further structure of the paper is the following: Section 2, Current research analysis, provides an overview of deep learning models of fake news classification. Section 3, Data, describes the data used for the experimental study. Section 4, Materials and methods, describes developed deep learning models. Section 5, Results describes the results of models' performance. Section 6, Discussion, discusses the classification results, perspective use of the models and their limitations. The conclusion describes the outcomes of the research.

## 2 Current research analysis

The proliferation of fake news in the digital age has necessitated the development of sophisticated tools and techniques for its detection and classification. Traditional methods of fake news detection, such as manual fact-checking and keyword-based approaches, have proven inadequate in dealing with the sheer volume and complexity of fake news circulating online (Cano-Marin et al., 2023). This has led to exploring machine learning and, more recently, deep learning models for fake news classification. Deep learning, a subset of machine learning, involves using neural networks with multiple layers (deep neural networks) to analyze various levels of data. These models have shown remarkable success in various natural language processing tasks, such as sentiment analysis (Mercha and Benbrahim, 2023), text summarization (Yousefi-Azar and Hamey, 2017), and language translation (Ali et al., 2021).

In the context of fake news classification, deep learning models have been employed to analyze the textual content of news articles and determine their integrity (Capuano et al., 2023). These models can process large amounts of data, extract intricate patterns, and capture the nuances of language, which are essential for accurately classifying fake news (Akter and Arora, 2023). Various architectures of deep learning models, such as Convolutional Neural Networks (CNNs), Recurrent Neural Networks (RNNs), and Long Short-Term Memory (LSTM) networks, have been explored for fake news classification. More recently, attention-based mechanisms, which allow the model to focus on the most relevant parts of the input text, have been incorporated into deep learning models to enhance their performance (Islam et al., 2020). These advancements in deep learning have contributed significantly to the ongoing efforts to combat fake news and paved the way for developing more accurate and efficient fake news classification models.

The paper Syed et al. (2023) addresses the challenge of fake news detection in the vast volumes of unlabeled data generated on social media platforms by proposing a hybrid approach that combines weakly supervised learning, deep learning, and feature extraction techniques. Specifically, the approach involves applying novel weakly supervised learning to provide labels to unlabeled data, followed by the use of Bidirectional Gated Recurrent Units (Bi-GRU) and Bidirectional Long Short-Term Memory (BiLSTM) deep learning techniques for fake news detection. Feature extraction uses Term Frequency-Inverse Document Frequency (TF-IDF) and Count Vectorizers techniques. The results indicate that the combination of BiLSTM and Bi-GRU deep learning techniques with Weakly Supervised Support Vector Machine (SVM) techniques achieved a 90% accuracy in detecting fake news, suggesting that the proposed approach is highly effective and efficient for fake and real news detection, especially when the data lacks labels.

The study Althubiti et al. (2022) focuses on designing and developing a novel model, Natural Language Processing with Sea Turtle Foraging Optimization-based Deep Learning Technique for Fake News Detection and Classification (STODL-FNDC), aimed at effectively discriminating fake news from legitimate news. The proposed STODL-FNDC model involves several steps: pre-processing of input data, Glove-based word embedding, and employing a Deep Belief Network (DBN) approach for detecting and classifying fake news. Subsequently, the Sea Turtle Foraging Optimization (STO) algorithm optimally adjusts the hyperparameters involved in the DBN model. The study's novelty lies in integrating the STO algorithm with the DBN model for Fake News Detection (FND). Simulations were conducted on benchmark datasets to enhance the detection performance of the STODL-FNDC technique. The experimental results demonstrated the superior performance of the STODL-FNDC approach compared to other methods, achieving a maximum accuracy of 95.50%. This indicates the effectiveness and efficiency of the proposed model in detecting and classifying fake news.

The study Abdulrahman and Baykara (2020) is centered on classifying fake news on social media, specifically focusing on textual content. This has become a crucial area of research due to the increasing preference for obtaining news on social media rather than traditional television, leading to a surge in fake content on these platforms. The study employed four traditional methods for feature extraction from texts: term frequency-inverse document frequency, count vector, character level vector, and N-Gram level vector. These features were then used to categorize the fake news dataset using 10 machine learning and deep learning classifiers. The results indicated that it is possible to classify fake news with textual content, mainly using a convolutional neural network. The study achieved an accuracy range of 81–100% using different classifiers, demonstrating the effectiveness of the proposed approach for fake news classification.

Dutta et al. (2022) proposes a hybrid deep learning classification model to identify and classify fake news and misleading information on the “COVID-19 Fake News Dataset” (taken from Mendeley), a collection of news or web articles related to COVID-19. The proposed classification model achieved an accuracy of 75.34%, outperforming existing LSTM and BiLSTM techniques. This demonstrates the effectiveness of the proposed model in automatically and accurately distinguishing between true and false information related to the COVID-19 pandemic.

The paper Ivancová et al. (2021) focuses on detecting fake news in articles written in the Slovak language. A labeled dataset of political news articles published by online news portals and suspicious conspiratorial portals was created to train deep learning models. Two architectures, CNN and LSTM neural networks, were trained using this data. The performance of the models was experimentally evaluated using standard classification metrics. The CNN model achieved an overall accuracy of 92.38%, with a recall metric of 95% for true news and 89% for fake news. Although both models are almost competitively balanced, the LSTM model is more suitable as it achieves higher overall accuracy and better recall values for both classes.

The paper Nordin et al. (2023) addresses the issue of fake news spread online, explicitly focusing on the Malay language. The study aims to evaluate the performance of a proposed Bidirectional RNN deep learning approach to classify fake Malay news by varying the dropout rate of the RNN model. Four different dropout values (0.1, 0.3, 0.5, 0.8) were used to evaluate the performance of the RNN models. The results indicated that a lower dropout rate required fewer epochs to train the RNN model, but the best accuracy (90.1%) was obtained with a dropout rate of 0.3. Higher dropout rates did not produce models with high accuracy values. The study concluded that maintaining a dropout percentage of 0.3 or below enables the LSTM to produce good accuracy values, and the length of the text highly influences the accuracy of the forecasted result. This study contributes to the field by providing a method for detecting fake news in Malay, which is currently under-researched.

The paper Alshahrani et al. (2023) addresses the issue of the spread of rumors or false information on social media platforms among Arab nations. The study develops a new hunter-prey optimization with a hybrid deep learning-based fake news detection (HPOHDL-FND) model on the Arabic corpus. The HPOHDL-FND technique involves extensive pre-processing steps to transform the input data into a valid format. It utilizes the LSTM-RNN model for fake news detection and classification. Finally, the hunter-prey optimization (HPO) algorithm is exploited to optimize the hyperparameters related to the LSTM-RNN model. The performance of the HPOHDL-FND technique was tested using two Arabic datasets, COVID-19 Fakes and satirical datasets. The results performed better than existing techniques, with a maximum accuracy of 96.57 and 93.53% on the COVID-19 Fakes and satirical datasets, respectively. This study contributes to the field by providing a novel and effective method for fake news detection in Arabic, a language for which fake news detection methods are critically needed.

Vo et al. (2022) addresses the challenge of fake news detection in Vietnamese. The authors present a tool developed to support fake news detection in Vietnamese by applying text classification techniques. A database was created consisting of four groups divided into two topics: politics and COVID-19, each further divided into fake news and real news. Deep learning techniques, CNN and RNN, were employed to create corresponding models. The tool classifies new news into one of the four groups to determine its authenticity. The tool detected fake news with a correct rate of about 85%, indicating that it could quickly and easily identify fake news. The authors suggest that this accuracy could be improved with a more extensive training dataset and by adjusting the machine learning model parameters. This work significantly contributes to fake news detection research for Vietnamese and can be applied to other languages. The authors also suggest that combining other methods, such as checking the source, verifying the author's information, and checking the distribution process, could improve fake news detection quality in the future.

The paper Ouassil et al. (2022) addresses the issue of detecting unreliable news spread through various online sources. The authors present a novel deep learning method for fake news detection, combining different word embedding techniques and a hybrid CNN and BiLSTM model. The classification model was trained on the unbiased WELFake dataset. The most effective method combined a pre-trained Word2Vec CBOW model and a Word2Vec Skip-Word model with CNN on BILSTM layers, achieving an accuracy of up to 97%. This result indicates the proposed method's high effectiveness in detecting fake news, contributing significantly to ongoing efforts to combat the spread of misleading information online.

The study Mouratidis et al. (2021) addresses the challenge of the rapid spread of fake news and propaganda on social networks. The authors present a novel approach for the automatic detection of fake news on Twitter, involving (a) pairwise text input, (b) a new deep neural network learning architecture allowing for flexible input fusion at various network layers, and (c) various input modes, such as word embeddings and both linguistic and network account features. Additionally, tweets are innovatively separated into news headers and news text, and classification tests are performed using both in an extensive experimental setup. The main results indicate high overall accuracy performance in fake news detection. The proposed deep learning architecture outperforms state-of-the-art classifiers, using fewer features and embeddings from the tweet text. This study contributes significantly to the ongoing efforts to combat the spread of fake news on social media platforms by proposing a novel and effective approach for fake news detection on Twitter.

Table 1 presents summary of deep learning models for fake news classification.

**Table 1:** Summary of deep learning models for fake news classification.

**References**	**Approach**	**Data source**	**Findings**
Syed et al. (2023)	Bi-GRU, BiLSTM	Twitter	BiLSTM and BiGRU with weakly supervised SVM shows the best performance for the classification of Fake news when compared to other state-of-the-art approaches using large amounts of weakly labeled data.
Althubiti et al. (2022)	STODL-FNDC	News articles	The proposed STODL-FNDC technique can be employed for effectual detection of fake news in real-time scenarios.
Abdulrahman and Baykara (2020)	ANN, RNN+LSTM, CNN+LSTM	Social media (text)	The study facilitated the use of machine learning and deep learning techniques at the same time on the same dataset, which provided insight into the capabilities of each classifier in classifying texts.
Dutta et al. (2022)	LSTM, BiLSTM, C-LSTM	News articles	The experiments demonstrated that C-LSTM-based deep learning model is more efficient than LSTM and BiLSTM models in COVID-19 Fake news classification.
Ivancová et al. (2021)	CNN, LSTM	News articles	LSTM architecture achieved superior performance, managing to detect most of the false articles while producing less false negatives as the CNN approach.
Nordin et al. (2023)	BiLSTM	News articles	Maintaining the percentage of dropout to be 0.3 and below enables the RNN model to produce good values of accuracies. The accuracy of the forecasted result also highly influenced by the length of the text.
Alshahrani et al. (2023)	LSTM-RNN	News articles	The HPOHDL-FND technique is tested using two Arabic datasets, and the outcomes exemplified better performance over the other existing approaches with maximum accuracy of 96.57 and 93.53% on COVID-19 fakes and satirical datasets, respectively.
Vo et al. (2022)	CNN, RNN	News articles	The solution based on text classification and deep learning suitable for fake news detection for Vietnamese news with a content analysis approach is proposed. Through testing, these tools correctly detected news as fake or real in about 85%.
Ouassil et al. (2022)	CNN, BiLSTM	News articles	The results show an improvement in terms of accuracy and precision when compared to traditional machine learning algorithms and related work results. The simple concatenation of the different pre-trained embedding models increases the dimension of embedded vectors.
Mouratidis et al. (2021)	CNN	Twitter	The study places high emphasis on the use of multimodal input that varies from word embeddings derived automatically from unstructured text to string-based and morphological features, and from higher-level linguistic features to network account-related features.

These findings underscore the potential of deep learning architectures, particularly those involving bidirectional LSTM, in fake news detection. Based on this foundation, our paper aims to develop a bidirectional LSTM and attention-based bidirectional LSTM architecture for fake news classification, contributing to the ongoing efforts to combat the spread of misleading information online.

## 3 Data

For the experimental study, we have used the WELFake open dataset (Verma et al., 2021a). The WELFake dataset is a comprehensive collection of news articles meticulously curated to provide a balanced and unbiased set of data, crucial for high-quality training data and delivering accurate results. While several open datasets are available for fake news study, these datasets have significant limitations in size, category, or bias. To address these limitations, the WELFake dataset was created by combining four existing datasets: Kaggle (Lifferth, 2018), McIntire (Hamel and Özkavci, 2023), Reuters (Shu et al., 2018), and BuzzFeed (Horne and Adali, 2017). This combination was chosen for two reasons: first, all four datasets have a similar structure with two categories, real and fake news; second, combining the datasets reduces the limitations and bias of each dataset. The resulting WELFake dataset comprises 72,134 news articles, classified as 35,028 real and 37,106 fake news articles. It contains three columns: title, text, and label, with a binary label for fake and real news.

Key observations from the dataset include:

- News articles containing between 450 and 550 words tend to be more reliable.
- General trends indicate that shorter, yet substantial news pieces are often more truthful.
- The text readability of fake news is poorer than the readability of real news.
- The subjectivity of fake news articles is more significant than real news articles.
- The number of articles representing real news is larger than those representing fake news.

These observations provide valuable insights into the characteristics of fake and real news articles, which can be instrumental in developing and refining fake news detection algorithms.

**Figure 1:**
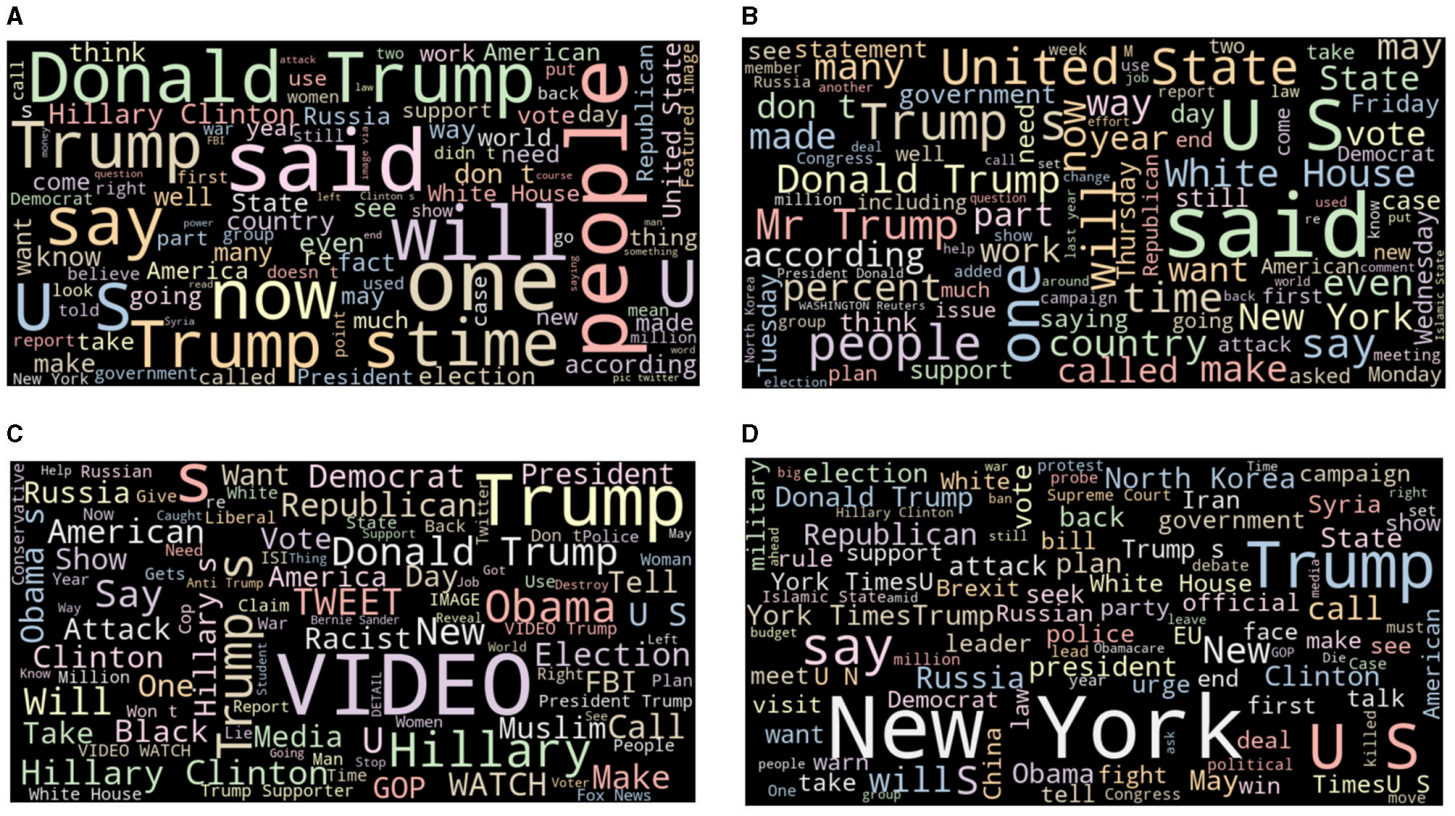
Dataset word cloud: **(A)** Fake text; **(B)** Real text; **(C)** Fake title; **(D)** Real title.

Figure 1 shows word cloud of the dataset most frequent words.

**Figure 2:**
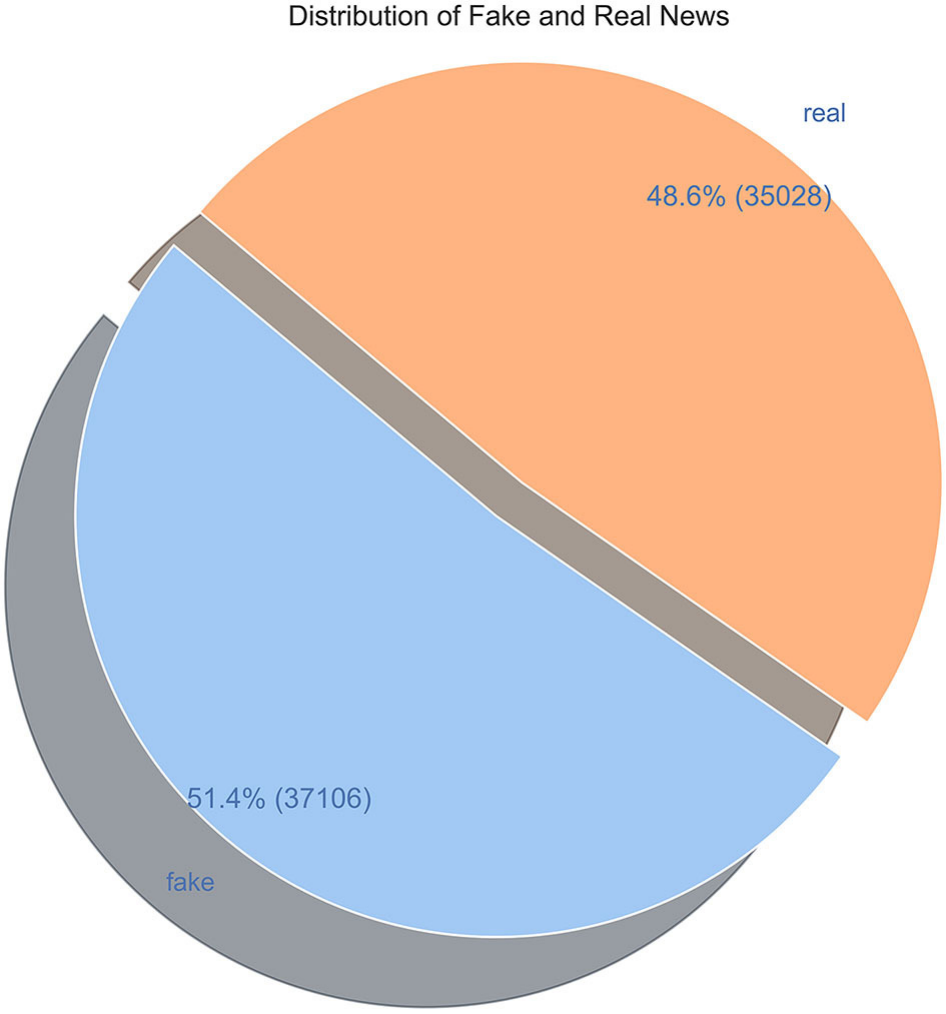
Dataset distribution.

Figure 2 shows the balanced distribution of fake and real news in the WELFake dataset.

**Figure 3:**
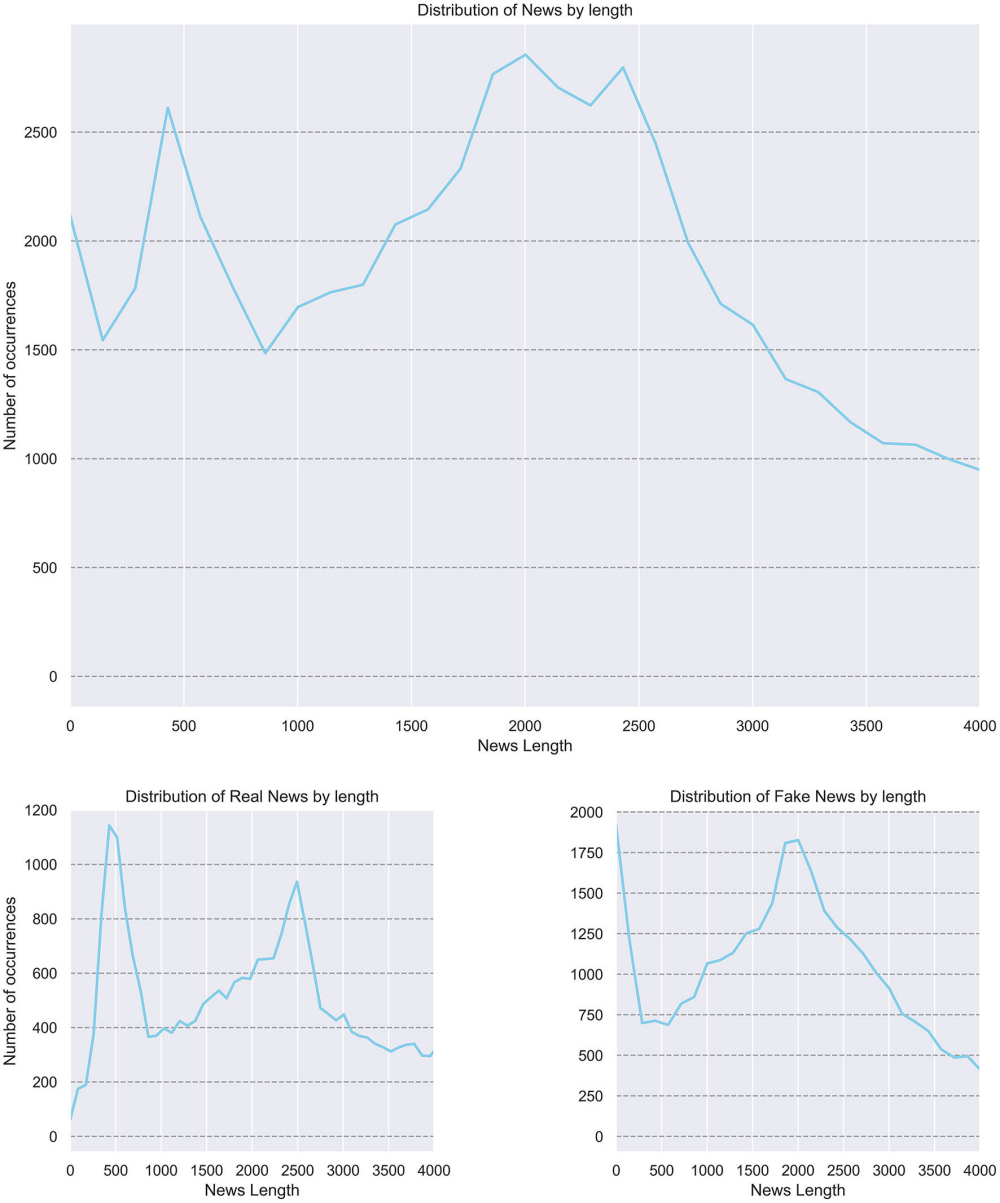
News lengths distribution.

Figure 3 shows news length distribution.

**Figure 4:**
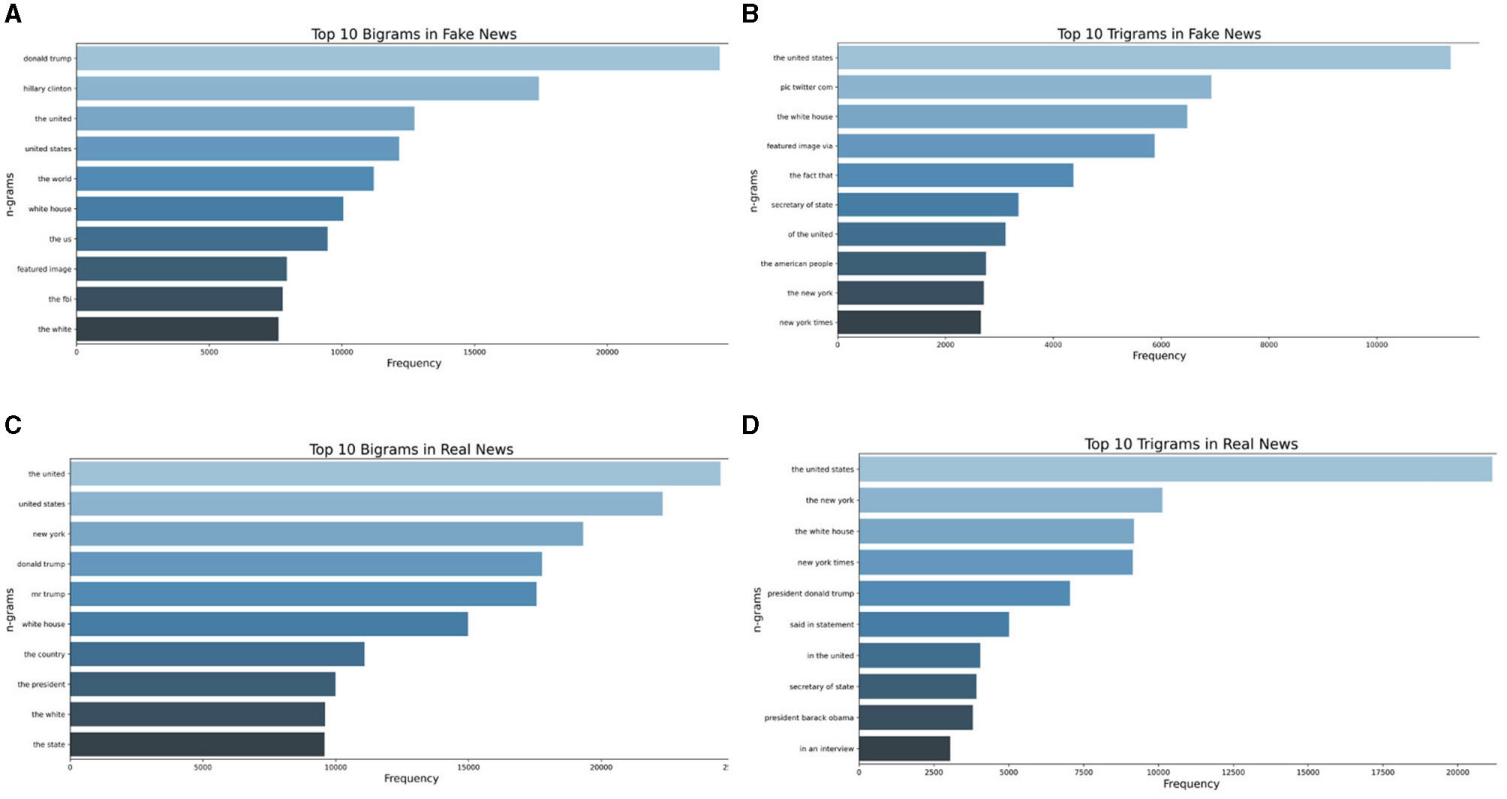
Dataset analysis: **(A)** Bigrams in fake news; **(B)** Trigrams in fake news; **(C)** Bigrams in real news; **(D)** Trigrams in real news.

Understanding the linguistic nuances and patterns inherent to deceptive narratives is crucial in the intricate landscape of fake news detection. Bigrams and trigrams, which represent sequences of two and three words, respectively, offer a granular perspective into the syntactic and semantic structures frequently employed in genuine and fabricated news articles. By analyzing these sequences, we aimed to capture the recurrent phrasal tendencies that indicate the veracity or falsehood of a news piece. Real news often adheres to a certain journalistic standard and style, which might manifest in specific word combinations. In contrast, fake news might exhibit recurrent patterns, potentially driven by sensationalism or other deceptive intentions. Describing and comparing the bigrams and trigrams of both categories provides a deeper linguistic insight, enabling a more robust and nuanced model for fake news classification. This approach enhances the model's accuracy and offers a tangible linguistic rationale behind its predictions, bridging the gap between computational methods and linguistic realities. Figure 4 shows the bigrams and trigrams of the dataset.

## 4 Materials and methods

### 4.1 LSTM

LSTM is a special kind of RNN capable of learning long-term dependencies in data (Hochreiter and Schmidhuber, 1997). Traditional RNNs suffer from the vanishing or exploding gradient problem, which makes it difficult for them to learn from data where past information is necessary to understand future data points (Levin, 1990). LSTMs were designed to overcome this limitation and are well-suited for classifying, processing, and making predictions based on time series data.

An LSTM network consists of memory cells arranged in a recurrent hidden layer, often referred to as units or nodes (Hochreiter and Schmidhuber, 1997). Each memory cell has three main components: an input gate, a forget gate, and an output gate, in addition to a cell state. These gates and the cell state work together to allow the LSTM to maintain or forget information over long data sequences:

1. Input gate. Determines how much new input should be added to the cell state. It consists of a sigmoid activation function that squashes the values between 0 and 1, and a tanh activation function that squashes values between −1 and 1. The sigmoid function decides which values to let through (0 means “let nothing through,” 1 means “let everything through”), and the tanh function gives the weightage to the values that are passed, which is then added to the cell state.
2. Forget gate. Determines how much of the current cell state should be forgotten or retained. It consists of a sigmoid activation function squashing values between 0 and 1. A value close to 0 means forget, and a value close to 1 means retain.
3. Output gate. Determines how much the current cell state should be output to the next layer. It consists of a sigmoid activation function that squashes the values between 0 and 1, and a tanh activation function applied to the cell state, squashing values between −1 and 1. The output is the multiplication of these two results.
4. Cell state. Represents the “memory” of the LSTM unit. It is a pathway that runs straight down the entire chain of LSTM units, with only minor linear interactions. The forget gate and the input gate update it.

At each time step, the LSTM unit receives an input, the previous hidden state, and the previous cell state:

1. The forget gate decides which parts of the cell state to forget.
2. The input gate decides which values from the input to update the cell state. The cell state is then updated by forgetting the specified parts and adding the new input.
3. The output gate decides which parts of the cell state to output as the hidden state for this time step.

**Figure 5:**
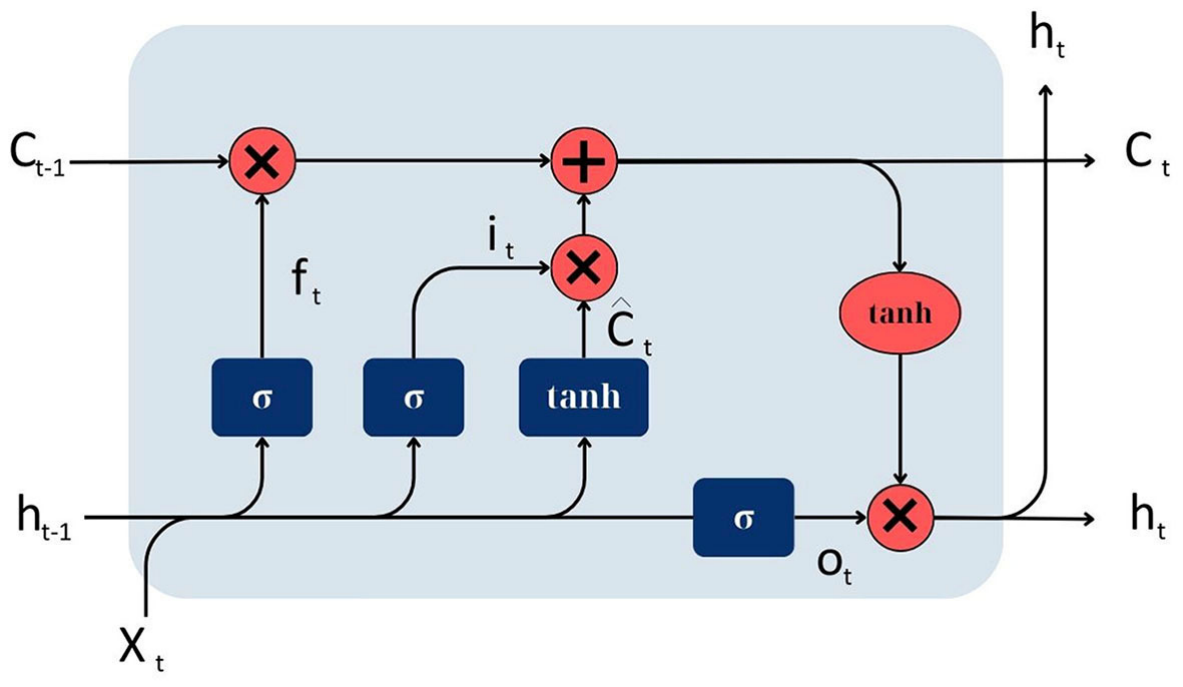
Architecture of LSTM model.

Figure 5 illustrates the structure of the LSTM model.

Advantages of LSTM model:

1. Capturing contextual information. Fake news often involves subtle cues and re-quires understanding the context over a sequence of words or sentences. LSTM models can capture long-term dependencies and contextual information in the text, which is crucial for accurately classifying fake news.
2. Handling variable length sequences. News articles can vary significantly in length. LSTMs can handle sequences of variable lengths, making them suitable for classifying news articles of different lengths.
3. Mitigating vanishing and exploding gradient problem. The vanishing and exploding gradient problem makes it difficult for traditional RNNs to learn from data where past information is necessary to understand future data points. LSTMs are resistant to these problems, making them more stable and effective in learning complex patterns in the text.

Disadvantages of LSTM model:

1. Computational complexity. LSTMs involve a complex structure with multiple gates and a cell state, increasing the model's computational complexity. This makes them computationally intensive and requires more time and resources to train, which can be a significant drawback for applications that require real-time classification of fake news.
2. Risk of overfitting. LSTMs have many parameters, which increases the risk of overfitting, especially when the available data is limited. This requires careful model design and techniques like dropout and regularization to mitigate this risk.
3. Interpretability. LSTMs, like other deep learning models, suffer from a lack of interpretability. It is often difficult to understand why the model makes a particular prediction. This can be a significant drawback for applications where interpretability is essential, such as fake news classification, where it may be necessary to understand and explain the reasons behind a classification.
4. Data dependency. The performance of LSTM models is highly dependent on the quality and quantity of the training data. If the training data is not representative of the actual data or insufficient training data, the model may not perform well. This is a significant challenge for fake news classification, as fake news is constantly evolving, and obtaining a representative and comprehensive dataset may be difficult.

### 4.2 BiLSTM

Bidirectional Long Short-Term Memory (BiLSTM) is a crucial architecture for the fake news classification task as it helps improve the model's performance on sequence classification problems (Graves and Schmidhuber, 2005). In fake news classification, all timesteps of the input sequence (the news article) are available; BiLSTMs train two LSTMs on the input sequence—the first on the input sequence as-is and the second on a reversed copy of the input sequence. Outputs at the same step from both LSTMs are then concatenated. This provides additional context to the network and results in faster and even fuller learning on the problem, which is essential for accurately classifying fake news (Zeng et al., 2019).

A BiLSTM consists of two LSTMs: one processing the input sequence (the news article) in a forward direction and another processing the input sequence backward. Each LSTM is a layer of recurrent units where each unit or node captures dependencies in the input sequence. The output of the two LSTMs is then concatenated and passed to the next layer.

1. Forward LSTM layer. This layer processes the input sequence (the news article) from the start to the end. It captures the contextual information from the past to the current timestep.
2. Backward LSTM layer. This layer processes the input sequence (the news article) from the end to the start. It captures the contextual information from the future to the current timestep.
3. Concatenation. The outputs of the forward and backward LSTM layers at each timestep are concatenated and passed to the next layer. This provides a complete view of the input sequence, capturing past and future contextual information at each timestep.

At each timestep, the forward LSTM processes the current input and the previous hidden state, while the backward LSTM processes the current input and the next hidden state. The outputs of both LSTMs are then concatenated and passed to the next layer. This allows the BiLSTM to capture past and future contextual information at each timestep, which is essential for accurately classifying fake news as it often involves subtle cues and requires understanding the context over a sequence of words or sentences.

**Figure 6:**
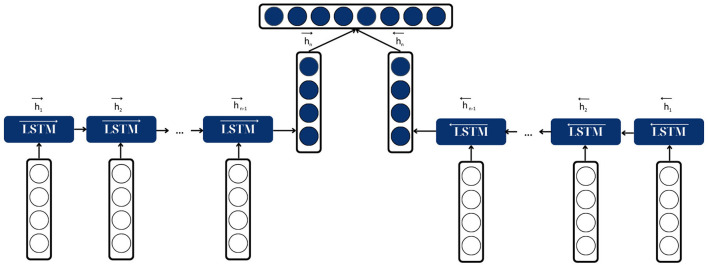
Architecture of BiLSTM model.

Figure 6 shows the architecture of BiLSTM model.

Advantages of BiLSTM model:

1. Capturing past and future context. BiLSTMs can capture past and future contextual information at each timestep, which is essential for accurately classifying fake news.
2. Better handling of long-term dependencies. By processing the input sequence (the news article) in both forward and backward directions, BiLSTMs can better capture long-term dependencies in the data, which is crucial for accurately classifying fake news as it often involves subtle cues and requires understanding the context over a sequence of words or sentences.

BiLSTMs are widely used in various applications, such as natural language pro-cessing, speech recognition, and time series prediction. They are particularly well-suited for the fake news classification task as they require capturing past and future context, such as machine translation, named entity recognition, and sentiment analysis.

**Figure 7:**
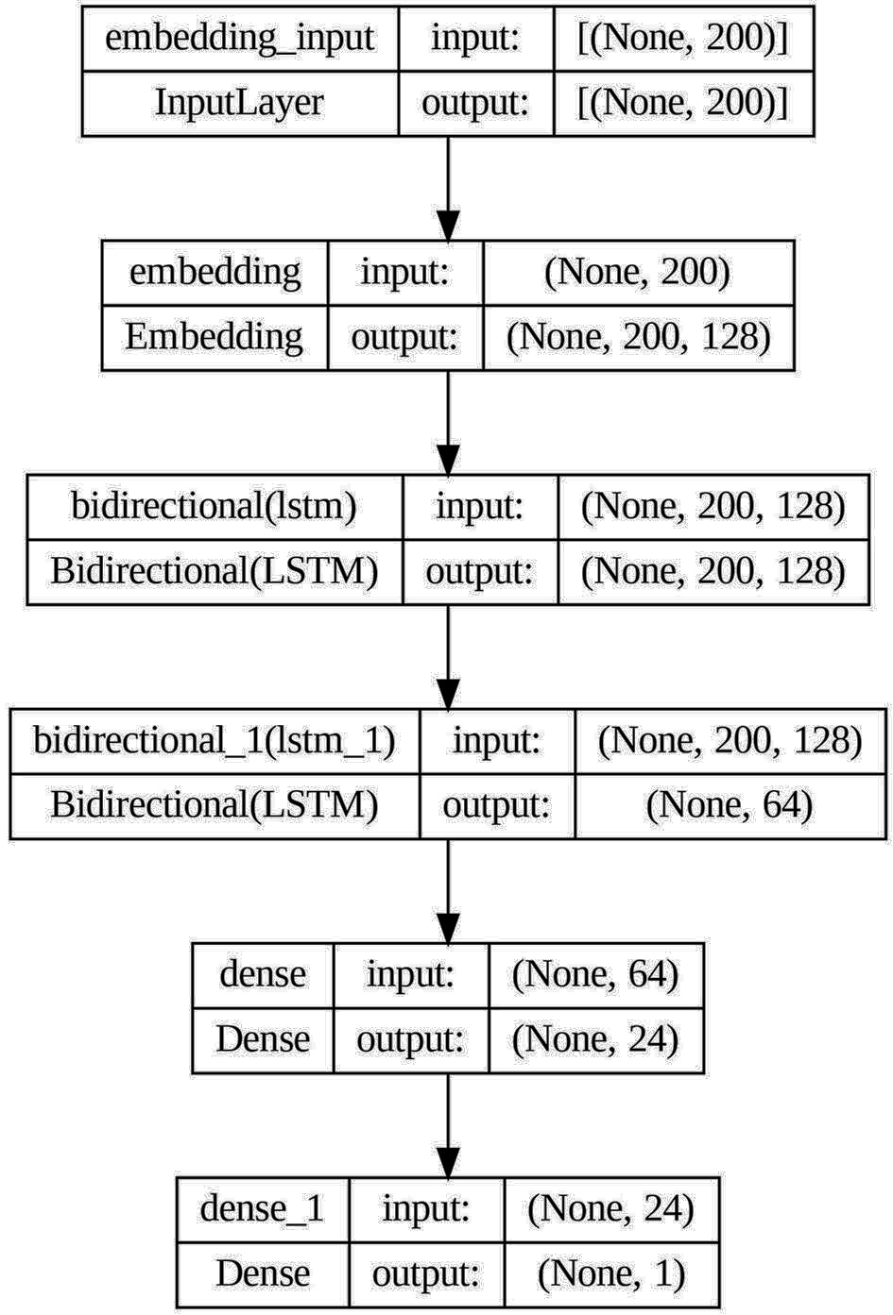
Proposed BiLSTM model architecture.

The proposed model architecture is shown in Figure 7.

### 4.3 Attention-based BiLSTM

The Attention-based Bidirectional Long Short-Term Memory (Attention-based BiLSTM) model combines the strengths of both the BiLSTM and the attention mechanism to create a more robust model for sequence classification tasks (Zhou et al., 2016).

The Attention-based BiLSTM model consists of three main components:

1. BiLSTM layer. This is the same as described in the previous response. It processes the input sequence (the news article) in both forward and backward directions and captures the past and future contextual information at each timestep.
2. Attention mechanism. This is a crucial component of the model. The attention mechanism allows the model to focus on different parts of the input sequence when producing an output sequence, essentially weighing the importance of different parts. In the context of fake news classification, the model can focus on the most important words or sentences in a news article that indicate it is fake or real.
3. Classification layer. This is the final layer of the model, which takes the weighted sum of the BiLSTM outputs (produced by the attention mechanism) and produces the final classification (fake or real).

The Attention-based BiLSTM model processes the input sequence (the news article) in the following steps (Chen et al., 2020):

1. The input sequence is passed through the BiLSTM layer, which processes the sequence in both forward and backward directions and produces a set of hidden states for each timestep.
2. The hidden states produced by the BiLSTM layer are then passed through the attention mechanism, which produces a weighted sum of the hidden states. This weighted sum is a single vector that summarizes the input sequence, with more important parts of the sequence receiving higher weights.
3. The weighted sum produced by the attention mechanism is then passed through the classification layer, which produces the final classification (fake or real).

**Figure 8:**
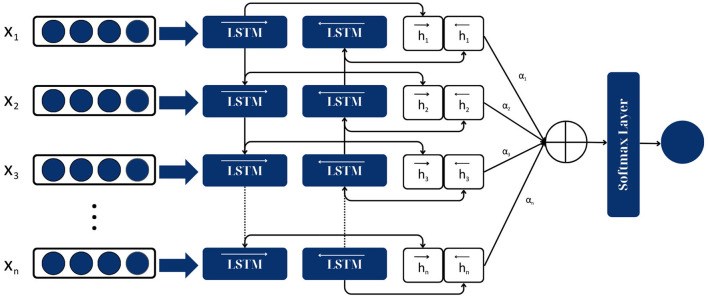
Attention-based BiLSTM model architecture.

The architecture of the attention-based BiLSTM models is shown in Figure 8.

Advantages:

1. Focus on important parts of the input. The attention mechanism allows the model to focus on a news article's most important words or sentences that indicate it is fake or real. This is crucial for accurately classifying fake news as it often involves subtle cues and requires understanding the context over a sequence of words or sentences.
2. Better handling of long-term dependencies. The BiLSTM layer allows the model to capture long-term dependencies in the input sequence, which is crucial for accurately classifying fake news.

The Attention-based BiLSTM model not only takes in information from the whole sequence but also has the ability to focus on its most important parts. This feature can enhance the effectiveness of the model.

**Figure 9:**
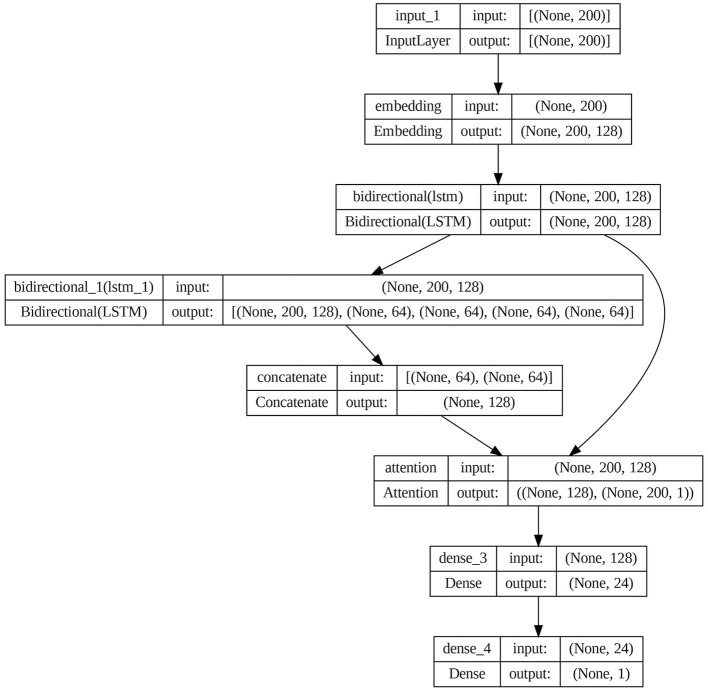
Architecture of proposed attention-based BiLSTM model.

The proposed model architecture is shown in Figure 9.

### 4.4 Performance evaluation metrics

In the context of fake news classification, several metrics are commonly used for evaluating the model's performance, each providing a view from different perspective.

The F1-score is the harmonic mean of precision and recall. It provides a single metric that balances the trade-off between precision and recall. It is calculated as:


$$\begin{array}{l} F1-Score\;=\;2\;\cdot \;\frac{Precision\cdot Recall}{Precision\;+\;Recall}. \end{array}$$

Accuracy is the ratio of correct predictions to the total number of predictions made. It is calculated as:


$$\begin{array}{l} Accuracy\;=\;\frac{True\;Positives\;+\;True\;Negatives}{Total\;Predictions}. \end{array}$$

While accuracy is a commonly used metric, it can be misleading in the context of fake news classification if the dataset is imbalanced (i.e., significantly more real news articles than fake news articles or vice versa).

Recall, also known as sensitivity or true positive rate, is the ratio of the number of true positive predictions (i.e., fake news correctly classified as fake) to the total number of actual positive instances (i.e., the total number of fake news articles in the dataset). It is calculated as:


$$\begin{array}{l} Recall\;=\;\frac{True\;Positives}{True\;Positives\;+\;False\;Negatives}. \end{array}$$

Recall is particularly important in the context of fake news classification, as it is crucial to identify as many fake news articles as possible to prevent their spread.

Precision, also known as the positive predictive value, is the ratio of the number of true positive predictions to the total number of positive predictions made by the model (i.e., the sum of true positives and false positives, where false positives are real news articles incorrectly classified as fake). It is calculated as:


$$\begin{array}{l} Precision\;=\;\frac{True\;Positives}{True\;Positives\;+\;False\;Positives}. \end{array}$$

Precision is important as it reflects the model's ability to correctly identify fake news articles without incorrectly classifying real news articles as fake.

Loss measures how well the model's predictions match the target values. Binary cross-entropy loss is commonly used for binary classification tasks like fake news classification. It is calculated as:


$$\begin{array}{l} Loss\;=\;-\frac{1}{N}\overset{N}{\underset{i=1}{\sum }}\left(y_{i}\;\cdot \;\log{\hat{y}}_{i}\;+\;\left(1\;-\;y_{i}\right)\;\cdot \;\log\left(1\;-\;{\hat{y}}_{i}\right)\right), \end{array}$$

where N is the number of samples, y_i_ is the actual target value for the i-th sample, and $\hat{y_{i}}$ is the predicted value for the i-th sample.

Each of these metrics provides a different perspective on the model's performance, and it is important to consider all of them when evaluating a model for fake news classification.

## 5 Results

The training process for the developed BiLSTM and attention-based BiLSTM models for fake news classification involved several key steps. First, the dataset was divided into training and testing sets, with 80% of the data used for training the model and 20% reserved for testing its performance.

The training data was then preprocessed, which involved tokenizing the text, removing stop words, and padding the sequences to ensure they all had the same length.

Next, the model was constructed. For the BiLSTM model, this involved creating layers for the embedding, bidirectional LSTM, and dense output. For the attention-based BiLSTM model, an additional attention layer was added between the BiLSTM and dense output layers. The model was then compiled, specifying the optimizer, loss function, and evaluation metrics for training. The model was then trained on the training data for a specified number of epochs, using a batch size that determined how many samples were used in each iteration to update the model's weights. During training, the model's performance was monitored on a validation set, a subset of the training data not used to update the model's weights. This helped to prevent overfitting and ensure that the model generalized well to new data.

The models' performance is shown in Table 2.

**Table 2:** Models' performance evaluation.

**Metric**	**BiLSTM**	**Att-BiLSTM**
F1-Score (test)	0.97481	0.97621
Accuracy (test)	0.97491	0.97657
Recall (test)	0.98406	0.97666
Precision (test)	0.96738	0.97702
Loss (test)	0.07437	0.07227
F1-Score (train)	0.98922	0.99205
Accuracy (train)	0.98927	0.99199
Recall (train)	0.98954	0.99254
Precision (train)	0.98968	0.99209
Loss (train)	0.03131	0.02527

The presented results offer a comparative analysis of the performance of two deep learning models, BiLSTM and Att-BiLSTM, for fake news classification based on various evaluation metrics.

Test Sample Analysis:

1. F1-Score: The BiLSTM model achieved an F1-score of 0.97481, while the Att-BiLSTM model had a marginally higher score of 0.97621.
2. Accuracy: Regarding overall accuracy, the BiLSTM model correctly classified ~97.49% of the test samples, while the Att-BiLSTM model had a slightly better accuracy of 97.66%.
3. Recall: The BiLSTM model achieved a recall of 0.98406, indicating it correctly identified ~98.41% of the actual positive samples. In contrast, the Att-BiLSTM model had a slightly lower recall of 0.97666 or 97.67%.
4. Precision: The precision for the BiLSTM model was 0.96738, suggesting that about 96.74% of the positive predictions were accurate. The Att-BiLSTM model slightly outperformed with a precision of 0.97702 or 97.70%.
5. Loss: The BiLSTM model registered a loss value of 0.07437, whereas the Att-BiLSTM model exhibited a slightly lower loss of 0.07227, indicating a marginally better model fit.

Training Sample Analysis:

1. F1-Score: The BiLSTM model had an F1-score of 0.98922, slightly lower than the Att-BiLSTM's score of 0.99205.
2. Accuracy: The BiLSTM model's accuracy was 0.98927 (98.93%), whereas the Att-BiLSTM model achieved a higher accuracy of 0.99199 (99.20%).
3. Recall: The BiLSTM model's recall was 0.98954 (98.95%), while the Att-BiLSTM model achieved a higher recall of 0.99254 (99.25%).
4. Precision: Both models showcased high precision on the training data, with BiLSTM at 0.98968 (98.97%) and Att-BiLSTM at 0.99209 (99.21%).
5. Loss: The BiLSTM model recorded a loss of 0.03131, while the Att-BiLSTM model demonstrated a lower loss of 0.02527.

While both models exhibited high performance on the training and test datasets, the attention-based BiLSTM model generally showed a slight edge in most metrics, especially on the training data. However, the differences between the two models' performances on the test data were marginal, suggesting that both models are robust and effective for fake news classification.

**Figure 10:**
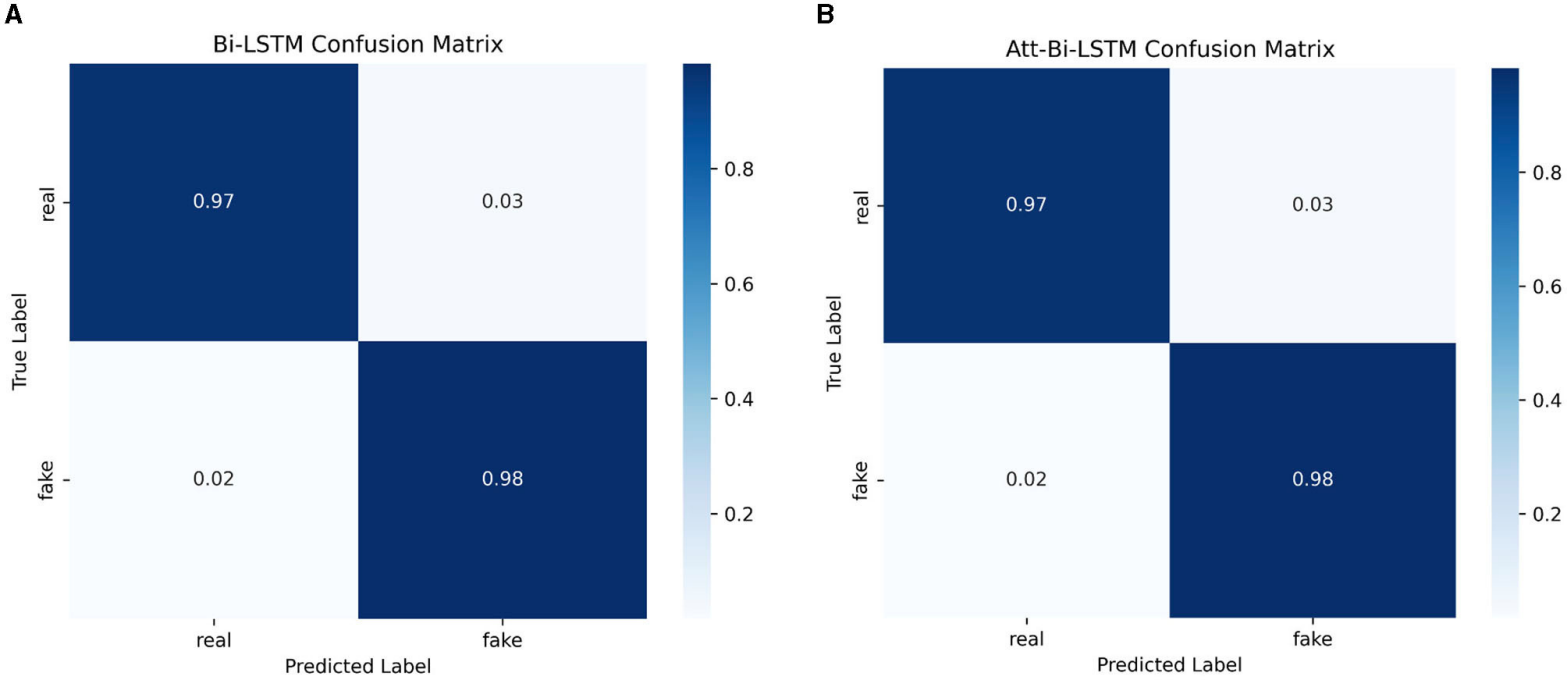
The confusion matrix for **(A)** BiLSTM model **(B)** attention-based BiLSTM model.

The confusion matrix for the both models is shown in Figure 10.

**Figure 11:**
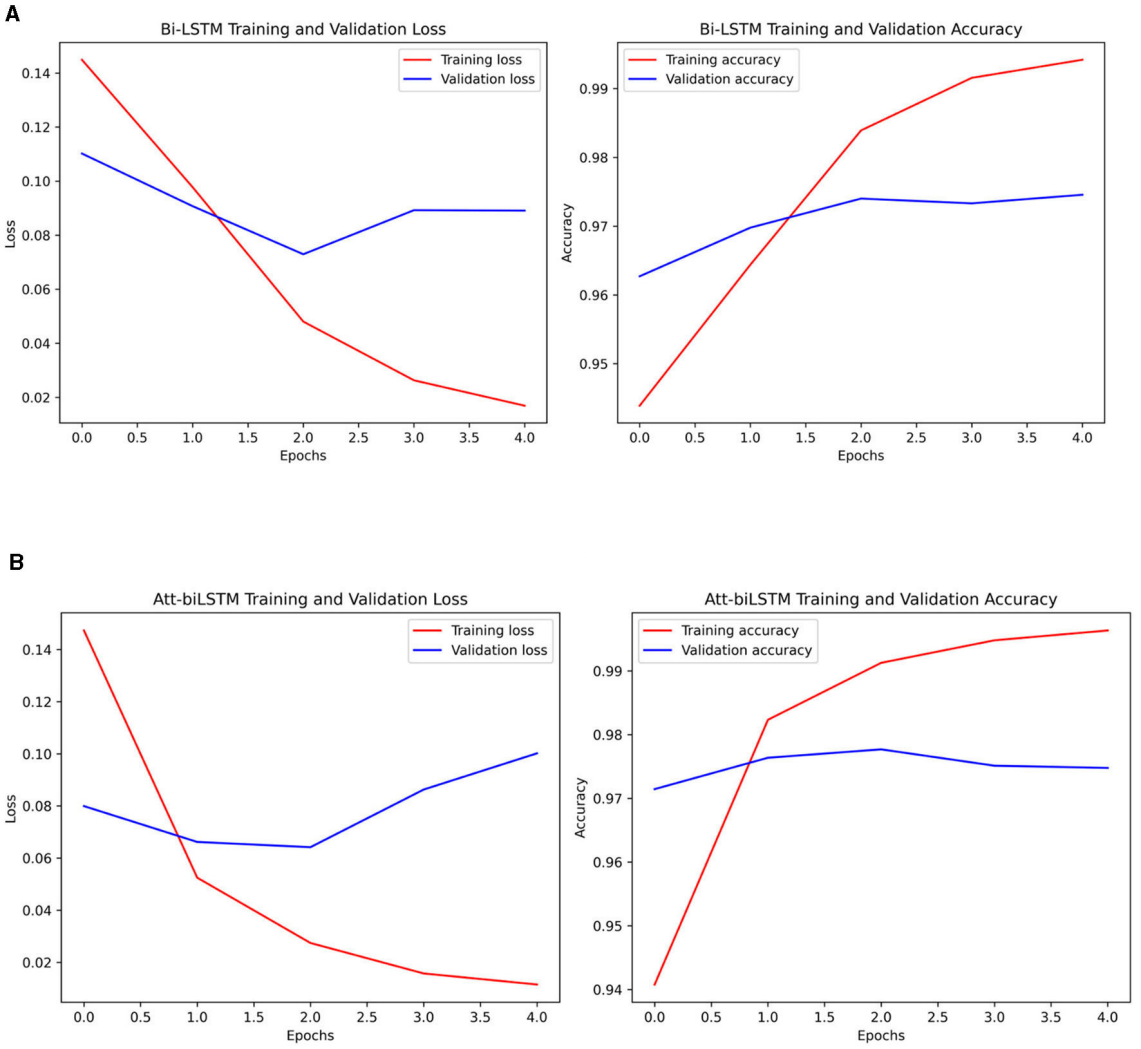
**(A)** BiLSTM and **(B)** Attention-based BiLSTM model loss and accuracy dynamics.

The dynamics of loss and accuracy for both models is shown in Figure 11.

**Figure 12:**
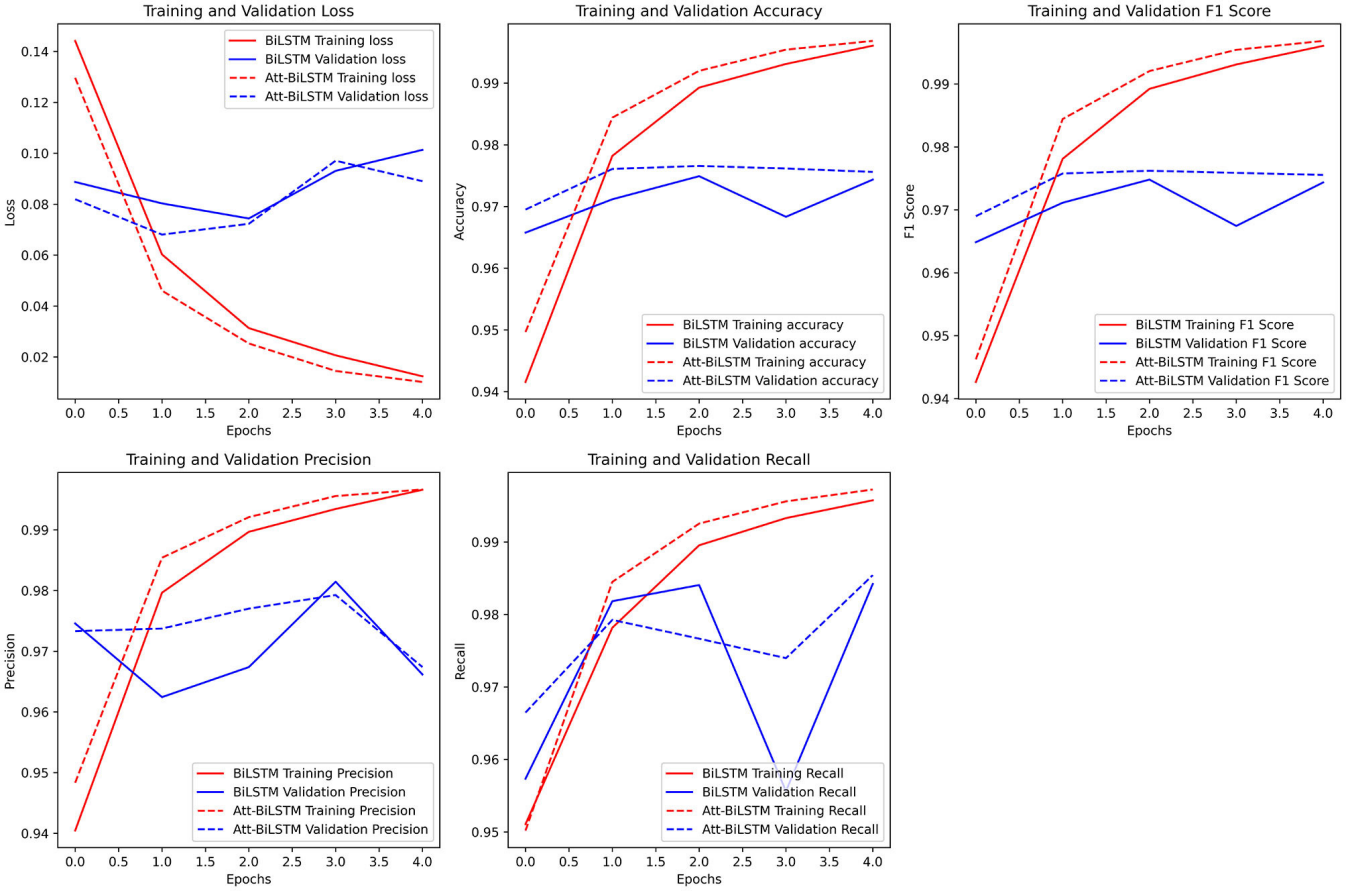
Comparison of models' performance.

The comparison of both models' performance is shown in Figure 12.

## 6 Discussion

The comparative analysis of the BiLSTM and attention-based BiLSTM models for fake news classification provides valuable insights into the efficacy of these deep learning architectures in identifying truthful news from fabricated content.

Both models demonstrated commendable performance on the test dataset. The BiLSTM model, with its recall of ~98.41%, showcased its strength in correctly identifying the most positive samples. However, the attention-based BiLSTM, despite a slightly lower recall, exhibited superior precision, suggesting fewer false positives. This precision is crucial in fake news detection, where falsely classifying genuine news as fake can have significant implications. The marginal difference in F1-score and accuracy between the two models indicates that both models provide a balanced trade-off between precision and recall. The loss values further corroborate the models' robustness, with attention-based BiLSTM having a slight edge.

The results from the training dataset underscore the models' capability to learn and generalize from the training data. Both models achieved high precision and recall values, with the attention-based BiLSTM model marginally outperforming the BiLSTM. The higher accuracy and lower loss of the attention-based BiLSTM model on the training data suggest its enhanced ability to fit the data without overfitting, given its performance on the test data.

The slightly superior performance of the attention-based BiLSTM model can be attributed to the integration of the attention mechanism. Attention mechanisms allow models to focus on specific parts of the input data that are more relevant to the task. In the context of fake news classification, the model can give more weight to specific phrases or patterns in the news content that indicate its authenticity. This nuanced approach might explain the attention-based BiLSTM's edge, especially in precision.

The results underscore the potential of deep learning models, particularly those with attention mechanisms, in fake news detection. Given the societal implications of unchecked fake news dissemination, the high performance of these models is promising. However, it is also essential to consider the slight performance variations between the models in different metrics, emphasizing the need to choose the suitable model based on the specific requirements of a fake news detection system.

Table 3 shows comparison of performance of the proposed models and other research, which used the same dataset.

**Table 3:** Models' performance comparison.

**Model**	**F1-score**	**Accuracy**	**Precision**	**Recall**
Att-BiLSTM (proposed)	0.9762	0.9766	0.9770	0.9767
BiLSTM (proposed)	0.9748	0.9749	0.9674	0.9841
N-Gram with TF-IDF and BERT (Kausar et al., 2022)	0.9630	0.9680	0.9650	0.9700
SVM (Verma et al., 2021b)	0.9656	0.9673	0.9460	0.9861
N-Gram with TF-IDF and LSTM (Kausar et al., 2022)	0.9580	0.9600	0.9550	0.9620
AdaBoost (Verma et al., 2021b)	0.9502	0.9532	0.9181	0.9846
Bagging (Verma et al., 2021b)	0.9500	0.9531	0.9178	0.9846
BiLSTM (Nirban et al., 2023)	0.9160	0.9200	0.9189	0.9131
Naïve Bayes (Verma et al., 2021b)	0.9185	0.9212	0.9145	0.9225
KNN (Verma et al., 2021b)	0.8978	0.9016	0.8902	0.9055
SVM (Nirban et al., 2023)	0.8974	0.9005	0.9216	0.8744
LSTM (Nirban et al., 2023)	0.8930	0.9015	0.9278	0.8607
Decision Tree (Verma et al., 2021b)	0.8924	0.8992	0.8610	0.9262
Memory-based ensemble model (Nirban et al., 2023)	0.8645	0.8730	0.8617	0.8672
NN with Keras (Nirban et al., 2023)	0.8579	0.8674	0.8637	0.8522
Non memory-based ensemble model (Nirban et al., 2023)	0.8531	0.8639	0.8837	0.8246
Random Forest (Nirban et al., 2023)	0.8329	0.8437	0.8362	0.8296
Naïve Bayes (Nirban et al., 2023)	0.7283	0.7589	0.8293	0.6492

The comparative evaluation of the proposed BiLSTM and attention-based BiLSTM models with other models from the literature provides a comprehensive understanding of the advancements in fake news classification.

Models such as KNN, SVM, Naïve Bayes, and Decision Tree (Verma et al., 2021b) exhibit varying performance degrees. While SVM shows a commendable accuracy of 96.73%, it still falls short compared to the proposed BiLSTM and Att-BiLSTM models. On the other hand, the Decision Tree and KNN models have relatively lower accuracy, emphasizing the limitations of traditional machine learning techniques in handling complex tasks like fake news detection.

Bagging and AdaBoost (Verma et al., 2021b) demonstrate competitive performance, with accuracies above 95%. However, their precision and recall metrics, especially compared to the proposed models, indicate room for improvement, particularly in minimizing false positives and negatives.

The paper Nirban et al. (2023) presents a range of neural network-based models, including standard LSTM, BiLSTM, and ensemble models. While these models, especially the BiLSTM from Nirban et al. (2023), show promising results, the proposed BiLSTM and attention-based BiLSTM models still outperform them in accuracy, precision, recall, and F1 score. This suggests the efficacy of the architectural improvements and optimizations made in the proposed models.

The models from the research (Kausar et al., 2022) that combine N-Gram with TF-IDF and advanced architectures like LSTM and BERT showcase high performance, with BERT achieving an accuracy of 96.80%. While these models are competitive, the proposed attention-based BiLSTM model slightly surpasses them.

The proposed BiLSTM and Att-BiLSTM models exhibit top-tier performance across all metrics. The attention based BiLSTM, with its attention mechanism, achieves an accuracy of 97.657%, making it one of the most effective models for fake news classification in the comparison. The high precision and recall values further underscore its capability to minimize false positives and negatives.

While the current results are encouraging, further research could delve into optimizing these models, exploring other attention mechanisms, or integrating additional features that could enhance the models' discerning capabilities. Additionally, understanding the models' performance across diverse datasets, including those in different languages or from varied sources, could provide a more comprehensive view of their applicability.

The BiLSTM and attention-based BiLSTM models have showcased their potential in the critical task of fake news classification. The slight advantages of the attention mechanism in the Att-BiLSTM model highlight the importance of model architecture choices in achieving optimal performance. As the digital information landscape continues to evolve, such deep learning models will play a pivotal role in ensuring the authenticity of the content consumed by the public.

The critical result of the paper is the analysis of Bi/Tri-grams of the dataset. Bi/Tri-grams serve as powerful tools in the realm of content analysis, offering a unique lens to decipher current trends and themes. By examining the most common word pairs or triplets, researchers can quickly identify patterns, popular subjects, and emerging narratives. It is important to mention that during analysis, we excluded phrases that lack informational significance on their own, such as “one of the,” “to,” “we are,” and “has been.”

An examination of the bigram chart for the fake dataset reveals that “Donald Trump” is the most prevalent phrase, appearing almost 25,000 times throughout the dataset. Ross and Rivers (2018) conducted a comprehensive study on Donald Trump's tweets, demonstrating his frequent use of derogatory labels like “fake news” and “fake media” to both express allegiance and mask his dissemination of misinformation presented as truth.

The frequent appearance of the phrase “Hillary Clinton” among the top bigrams indicates that, despite her electoral defeat, Clinton remains a highly mentioned politician and a primary adversary of then-incumbent President Trump. This is particularly relevant in the context of the 2018 midterm elections in the USA, which were rife with misinformation targeting representatives from both the Republican and Democratic camps.

Other top bigrams, such as “United States” and “white house,” are relatively generic, signifying the citizens' deep engagement in nation-building processes like elections in their country. Interestingly, the bigrams for real news do not differ significantly from those of fake news regarding the top 10 phrases. The only notable absence is the phrase “Hillary Clinton.” However, the general nature of the words reflects political processes in the context of the USA's midterm elections, with terms like “Donald Trump,” “the president,” “the country,” “United States,” “White House,” and their variations.

The presence of “New York Times” and “The New York” in the trigrams of the fake set suggests frequent references to this media outlet in the news. In fake news, this trigram occurs only 2,700 times, whereas in true ones, it occurs 10,000 times. It is plausible that the fake news sample often cited this source to enhance its credibility. According to a 2018 Gallup Institute survey, ~64% of respondents perceived information published in the NYT as a highly accurate, very accurate, or somewhat accurate source (Watson, 2018).

The appearance of phrases like “pic Twitter com” and “featured image via” in the fake news trigram rankings indicates that misinformation from social networks often permeates online media. This suggests that media outlets may rely on less credible sources from social networks, referencing their illustrations and graphics.

This approach is not to be compared in terms of superiority or inferiority with machine learning; rather, it complements it. While machine learning models delve deep, deciphering intricate relationships and predicting patterns, Bi/Tri-grams provide a more immediate, surface-level insight. Both methods offer their distinct advantages, with Bi/Tri-grams providing a straightforward snapshot of content trends, whereas machine learning offers a deeper, more nuanced understanding. Together, they form a holistic approach to understanding content in its many layers.

While promising, the study on fake news classification using Bi-LSTM and attention-based Bi-LSTM models presents certain limitations that warrant consideration. One limitation is the dependency on data quality and representativeness. The effectiveness of the models is closely tied to the diversity and real-world applicability of the training data. In scenarios where the training data lacks variety or fails to capture the nuances of real-world fake news, the models may struggle to generalize effectively to new, unseen data. Another concern is the potential for overfitting, a common challenge in deep learning models with many parameters. Despite implementing measures like dropout to mitigate this risk, the possibility of the models fitting too closely to the training data and not performing well on new data remains a pertinent issue. Additionally, the study's focus on specific languages and contexts implies that the models' effectiveness in other linguistic or fake news dissemination scenarios is yet to be established. Extending the applicability of these models to a broader range of languages and contexts is crucial for their utility in diverse fake news classification tasks. Addressing these limitations in future research is essential to enhance the robustness and wider applicability of fake news classification models.

Future research directions in fake news classification, particularly using deep learning models like Bi-LSTM and attention-based Bi-LSTM, offer a rich landscape for exploration and innovation. One promising avenue is the enhancement of data diversity and representativeness. Future studies could focus on curating more comprehensive datasets encompassing a wider range of fake news examples, including those from different languages, cultures, and digital platforms. This expansion would test the models' robustness and adaptability and ensure their applicability in a global context where fake news has no linguistic or cultural boundaries.

Another significant area for future research lies in refining model architecture and efficiency. While the current models demonstrate high accuracy and effectiveness, there is always room for improvement in computational efficiency and processing speed. This could involve exploring lighter model architectures that maintain high accuracy while being more resource-efficient, making them suitable for deployment in environments with limited computational resources. Additionally, integrating multimodal data, such as images or videos accompanying text, could provide a more holistic approach to fake news detection, as fake news often comprises complex combinations of various media types.

The practical use of the proposed models extends beyond academic research into media, politics, and public information dissemination. Media organizations can employ these models to automatically filter out fake news from genuine articles, thus maintaining the credibility and trustworthiness of their content. In the political sphere, these models can be instrumental in identifying and countering misinformation campaigns, thereby safeguarding the integrity of democratic processes. Integrating such models into social media platforms and news aggregators for the general public can provide a first line of defense against the spread of misinformation, empowering users to make informed decisions based on reliable information.

The path forward for research in fake news classification using deep learning models is challenging and exciting. It promises more sophisticated, efficient, and globally applicable models that can significantly contribute to the fight against misinformation, ultimately fostering a more informed and truthful digital information landscape.

## 7 Conclusions

The digital age, characterized by the rapid dissemination of information, has brought with it the challenge of discerning genuine news from fake narratives. This study was conceived to address this pressing issue, focusing on developing and evaluating innovative deep learning models, specifically the BiLSTM and attention-based BiLSTM architectures, for the task of fake news classification.

A comprehensive suite of evaluation metrics was employed, encompassing Recall, Precision, F1-Score, Accuracy, and Loss. These metrics ensured a multi-faceted evaluation, capturing the models' precision, recall, and accuracy in classifying news articles.

The proposed models demonstrated superior efficacy when benchmarked against a range of models from existing literature, both from traditional machine learning and advanced deep learning paradigms. The attention-based BiLSTM, in particular, emerged as a frontrunner, highlighting the advantages of combining attention mechanisms with LSTM structures.

The dataset was split, with 0.8 of data allocated for training the models and 0.2 reserved for testing them. The performance on the validation set was instrumental in fine-tuning the models, while the test set results offered an objective assessment of their real-world performance.

Research marks a significant advancement in fake news detection's scientific and practical realms. Scientifically, the novelty of this research lies in the sophisticated application of deep learning models, specifically the Bi-LSTM and attention-based Bi-LSTM architectures, tailored for the nuanced task of distinguishing authentic news from fabricated stories. This approach represents a notable shift from traditional methods, offering a deeper understanding of fake news's linguistic patterns and subtleties. Integrating the attention mechanism within the Bi-LSTM framework is particularly innovative, as it enables the model to focus selectively on the most informative parts of the data, thereby enhancing the accuracy and reliability of classification.

Practically, the study's novelty is evident in its direct applicability to real-world scenarios. The developed models provide robust tools for media outlets, social media platforms, and information verification agencies to filter out fake news automatically and efficiently. This capability is crucial in an era where the rapid spread of misinformation can have far-reaching consequences on public opinion, political processes, and societal trust. By offering a high degree of accuracy in fake news detection, these models can significantly contribute to maintaining the integrity of information dissemination across digital platforms. The practical implications of this research extend to enhancing the quality of information consumed by the public, thereby fostering a more informed and discerning society.

Beyond its immediate findings, this study paves the way for future research in misinformation detection. The demonstrated potential of deep learning, especially the novel architectures proposed here, underscores the vast possibilities in Natural Language Processing. Subsequent studies could delve deeper into integrating these architectures with other advanced models or explore multi-modal fake news detection, encompassing visual and auditory data.

Summarizing, this research has provided effective tools for fake news detection and introduced novel methodologies that set new standards in the field. As we navigate the complexities of the digital information era, such innovative approaches will be instrumental in preserving the authenticity of information and upholding the sanctity of public discourse.

## Data availability statement

The original contributions presented in the study are included in the article/supplementary material, further inquiries can be directed to the corresponding author.

## Author contributions

HP: Conceptualization, Data curation, Formal analysis, Investigation, Methodology, Project administration, Validation, Visualization, Writing – original draft. VC: Conceptualization, Data curation, Investigation, Methodology, Software, Validation, Visualization, Writing – review & editing. DC: Conceptualization, Data curation, Formal analysis, Funding acquisition, Investigation, Resources, Supervision, Writing – original draft.
